# Re-tooling critical care to become a better intensivist: something old and something new

**DOI:** 10.1186/cc14721

**Published:** 2015-12-18

**Authors:** John J Marini

**Affiliations:** 1 Department of Medicine, University of Minnesota, Regions Hospital 11203B, 640 Jackson St., St. Paul MN 55101, USA

## Abstract

Developments in recent years have placed powerful new tools of diagnosis, therapy, and communication at the disposal of medicine in general, and of critical care in particular. The art of healing requires not only technical proficiency, but also personal connection, multidisciplinary teamwork, and commitment to the venerable traditions of our profession. The latter often seem to be under assault by today's high-pressure, high-efficiency, and increasingly business-driven hospital environments. Re-tooling critical care for the future generations of caregivers requires something old--empathetic connection--as well as the exciting newer technologies of our science and practice.

## Introduction

From a historical perspective, the organized discipline of critical care medicine is a relatively young field. Intensive care practice initially grew out of a tradition of anesthesiology, where principles of life support learned in the operating theater were extended to the protracted recovery care of recently operated patients and eventually to others needing life support for extended periods of time. The first ICUs were developed in the United States in the 1960s, but formal certification in critical care medicine was not offered in our discipline until two decades later [1]. As our field matured, we learned to rescue many patients who otherwise would have succumbed to catastrophic illness. We have improved the processes of care delivery and better understood the nuances of many critical illnesses and their management. Indeed, by the simple standard of survival alone, our progress has been impressive. For example, the incidences of acute lung injury and acute respiratory distress syndrome (ARDS) have substantially and steadily declined [2], and survival from sepsis is reported to have improved from where it was only a few years ago [3]. But the news for our field has not all been good. Along the way, one of the key lessons learned is that our well-intentioned treatments, such as excessive sedation and aggressive ventilatory patterns, may themselves lead to disability and injury. Whereas our progress in physiological understanding and reduced iatrogenic injury is undeniable, practitioners have now become concerned that our methods may themselves be resulting in lasting disability that extends well beyond the temporal boundaries of the ICU experience [4,5]. The desire to reduce chronic critical illness and disability that result unintentionally from our cares has become a major impetus for current research [6].

Apart from its scientific underpinnings and technical improvements, our field has changed dramatically in other ways, some of which continue to erode the traditional and venerated core principles of medicine, especially that of the patient-physician relationship. The physician has traditionally occupied a unique role in society--one of healer, councilor, adviser, advocate, as well as technically proficient practitioner. Three vital tenets of traditional practice have been strong connection to the patient and family, clear communication, and heartfelt compassion. The latter characteristic--perhaps the most cherished of those qualities that distinguish the excellent from the merely competent caregiver--is difficult to nurture in today's high-paced critical care environment [7]. To understand what has happened, and thereby to gain insight into how this challenging field may be profitably redirected, it is useful to understand the historical basis for certain fundamental changes that have driven us to our current reality. Having entered the field just as it was beginning to gain momentum in the late 1970s and having been carried along by powerful currents of change, my own perspective may be instructive regarding what transpired. At the outset it must be understood that what follows are my own perceptions, which undoubtedly differ from those of others and admittedly are open to debate.

Faced with a difficult choice of selecting a specialty after completing a basic broad education in internal medicine, I was drawn toward intensive care for several reasons. I chose to enter this difficult field precisely because it was wide ranging, dynamic, exciting, and challenging. Intensive care promised to afford the opportunity to apply science and logic to problems of unquestioned importance to the individual patient. Moreover, I had strong academic role models to tangibly demonstrate that the academic pathway was an achievable blend of research, patient care, and education. Back then, our still young discipline had not yet approached a functional plateau, but was steadily developing, highly malleable, and shaped by strong but in some ways subtle forces that I, for one, did not take into consideration. Three important categories of these driving forces were: evolving technology; social changes that dramatically affected the composition and priorities of the physician workforce; and the rising importance of economic considerations to academic institutions as well as to privately practicing caregivers. Although each force category has exerted strongly positive impacts, the negative consequences of their changes have also been felt. Together they have encouraged fragmentation of care and a resulting disconnection of the physician from the patient, the nurse, and traditional professional ideals. In what follows I will expand selectively upon these negative aspects, which again are highly personal in their interpretations.

Adverse changes strongly affected science, education, and patient care--the three legs of the traditional academic footstool. In science, our field improved rescue and support methodologies, benefiting markedly from exotic new tools, monitoring equipment, and techniques. Starting in the early 1980s and continuing to the present time there has been inexorable movement toward population-based research, as exemplified by the evidence-based medicine (EBM)/randomized clinical trial (RCT) orientation [8]. Strong movement also pushed funding of science toward molecular mechanisms and genetics, with the laudable intentions of discovery of mechanisms and interventions closer to the fundamental biology of disease [9,10]. Simultaneously, however, there was a movement away from system physiology and pathophysiology and away from clinical observation and experimentation. Together, these scientific forces encouraged separation of the academic physician/caregiver from the patient's bedside and from daily clinical problems. Funding for research, the lifeblood of academic activity, was progressively diverted from animal research and observational studies of problems encountered in everyday practice to those based in epidemiology, molecular science, and drug discovery. As an unintended consequence, the traditional academic role model of clinician-scientist has all but disappeared from our training environments [11].

Concerning sea changes also occurred over this same period regarding education. Expenses associated with undergraduate and medical education have soared, increasing dramatically faster than the general cost of living. Many young professionals now graduate with crushing debt to repay [12]. The physical demands and consequences of long work hours and sleep deprivation were openly recognized [13]. With the entry of many more women into the medical field as well as into other stressful, high-level professions, decisions regarding timing of family initiation and issues of deferred child-rearing had to be confronted. Married householders often found that traditional family roles would not withstand the rigors of two time-consuming careers without serious revision; the number of hours spent at the hospital dedicated to training and practice could not be left open ended. Mandates for fewer training hours emerged and were strongly supported, partially in response to strident calls for rebalancing work and personal time [13]. Both the trainee and the practitioner were challenged by the demands of more to know and more to document with less time to allocate for them both. Saddled by debt and unpersuaded by the value and feasibility of an academic career, most trainees increasingly sought secure, well-paying, and lifestyle-consistent positions rather than the tough, uncertain, and less remunerated academic route.

Electronic health records (EHR) and computerization of the care delivery environment have unquestionably aided us in retrieving and sharing detailed information relevant to the specific patient and in rapidly retrieving diagnostic and therapeutic information from the digitized literature [14,15]. Improved information retrieval and online learning have also improved the efficiency (if not always the outcome) of our educational efforts. Scripted conferences and simulations have complemented, and in many instances have gradually taken the place of, clinical exposures and mentored real-time experience with medical procedures. Such trends have had both strongly positive and negative impacts. From the standpoint of a bedside teacher, there seems to have been palpable erosion of critical and analytical thinking skills as students and residents prioritize labeling of conditions and following prepackaged care protocols. Emphasis is placed on what to do, rather than on deliberating such questions as "How does this observation or consideration fit into the clinical picture?" or "Is this plan or decision logical or not?" Such a "label and look-it-up" mentality seems now poised to replace sound reasoning.

Providing patient care is intrinsically rewarding. Yet a considerable proportion of that derived satisfaction is rooted in traditionally close patient-physician relationships. The enormous changes of the past few decades have also shaken this treasured connection. Improved knowledge and broadened options for intervention have raised expectations and spawned increased demands for service from patients and delivery systems alike. Answering such calls for service has been encouraged and well remunerated by the reorganized care delivery systems we now work within. Compared with the early years of critical care, both nurse and physician are better compensated. Responding to economic incentives and competition, business models have preempted traditional physician-driven care delivery while competitive expansion has diluted regional expertise. Subspecialization and hospitalist movements have gained traction, so that multiple caregivers, who are unfamiliar to the patient and who attend to a large panel of cases, transiently engage the critically ill individual but quickly migrate to the next site of action. Electronic medical records (EMRs) have also encouraged a retreat from the bedside, so that less physician time is spent in physical proximity to the patient and nurse [14,15]. Sadly, there has been a subtle but undeniable erosion of patient trust in this expensive and depersonalized medical system, damage that has encouraged litigation for unanticipated clinical results. Thus, from my perspective, underlying economic forces have provided well-appreciated and well-deserved financial remuneration but also have encouraged many of the undesirable changes occurring in our field. To coin a new corollary to a very familiar aphorism, might I suggest: "Money may not be the root of all evil, but it seems to be a very good fertilizer"?

Satisfaction with medical practice varies from one caregiver to the next. It is undeniable, however, that intrinsic satisfactions which were in place three decades ago for the field of critical care have been eroded and continue under inexorable assault. Under pressure to perform more work and devote less time per patient, stress and fatigue alter caregiving attitudes and priorities. Physicians are under time pressure to complete their work and to document billable charges before the scheduled end of the workday. Such pressures often result not only in suboptimal medical care but also in compassion fatigue and professional burnout [16,17].

## Addressing the problems

**Figure 1:**
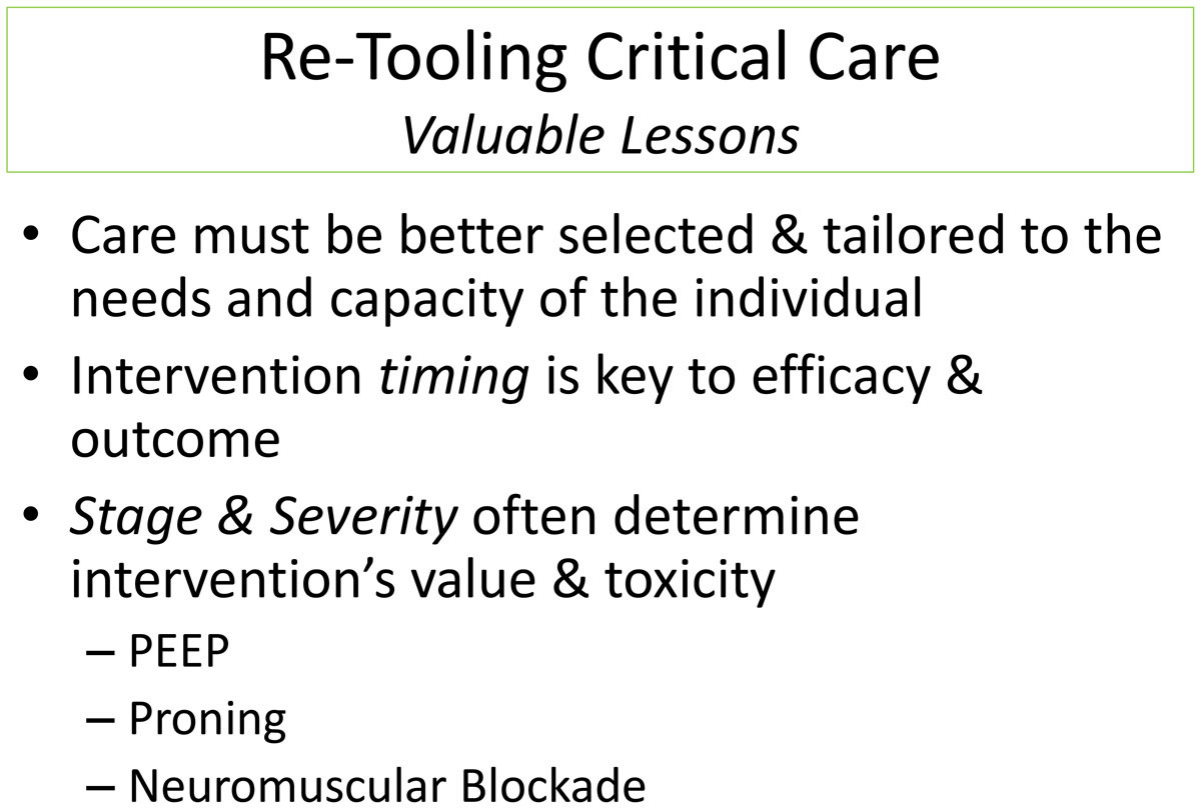
**Re-tooling critical care: *valuable lessons***.

As most experienced clinicians acknowledge, RCTs in critical care help set general directives and raise important questions but alone cannot reliably guide decisions made for the individual. Patient diversity coupled with imprecise disease definitions and failure of flawed study designs to account for differences in comorbidities, cointerventions, and varied care delivery environments render RCT conclusions questionably applicable to specific situations. Moreover, because medical practice changes continually, the results of any RCT are questionably relevant to a newer clinical context. Appropriate ICU care requires logic and analytical ("critical") thinking. Protocols and rapid access to relevant information help considerably, but are not enough to deliver excellent care. When facing a complex and changing problem, application of the set of versatile tools provided by deep physiological understanding remains the appropriate approach. It is precisely these skills that need to be refreshed in our current medical educational environment (Figure 1).

Personal characteristics that have become less evident in today's clinical setting may help restore vital links between doctor and patient. Our students and residents must take ownership of their cases rather than reject primary responsibility for their management decisions and consequences. Embracing responsibility requires follow-up and concerned follow-through, even after the patient has left the ICU. The dedicated ICU physician stays curious, thinks for himself/herself, and plans two steps ahead of the situation of the moment; a proactive rather than reactive orientation is to be encouraged. Simultaneously, the caregiver must not always follow the crowd that adopts new therapies or discards the old without careful deliberation. Rather than rushing through the day, the excellent ICU physician stays objective, listens attentively, and reflects on new data of relevance. Integrating and continually updating all streams of useful information is fundamental to scientific practice. These information sources not only include the patient's current imaging, laboratory data, and medication information, but also awareness of the patient's physiological reserves, the medical history, and prior responses to therapy, both with respect to tolerance and toxicity. Family discussions often prove instrumental for understanding the current presentation. The better caregiver is quick to consult the literature in order to obtain deeper understanding of unfamiliar problems. Before committing to interventions with the potential for harm, such as surgical operations and routine bedside procedures, a thoughtful physician "measures twice and cuts once" so as to avoid unintended harm and expense.

**Figure 2:**
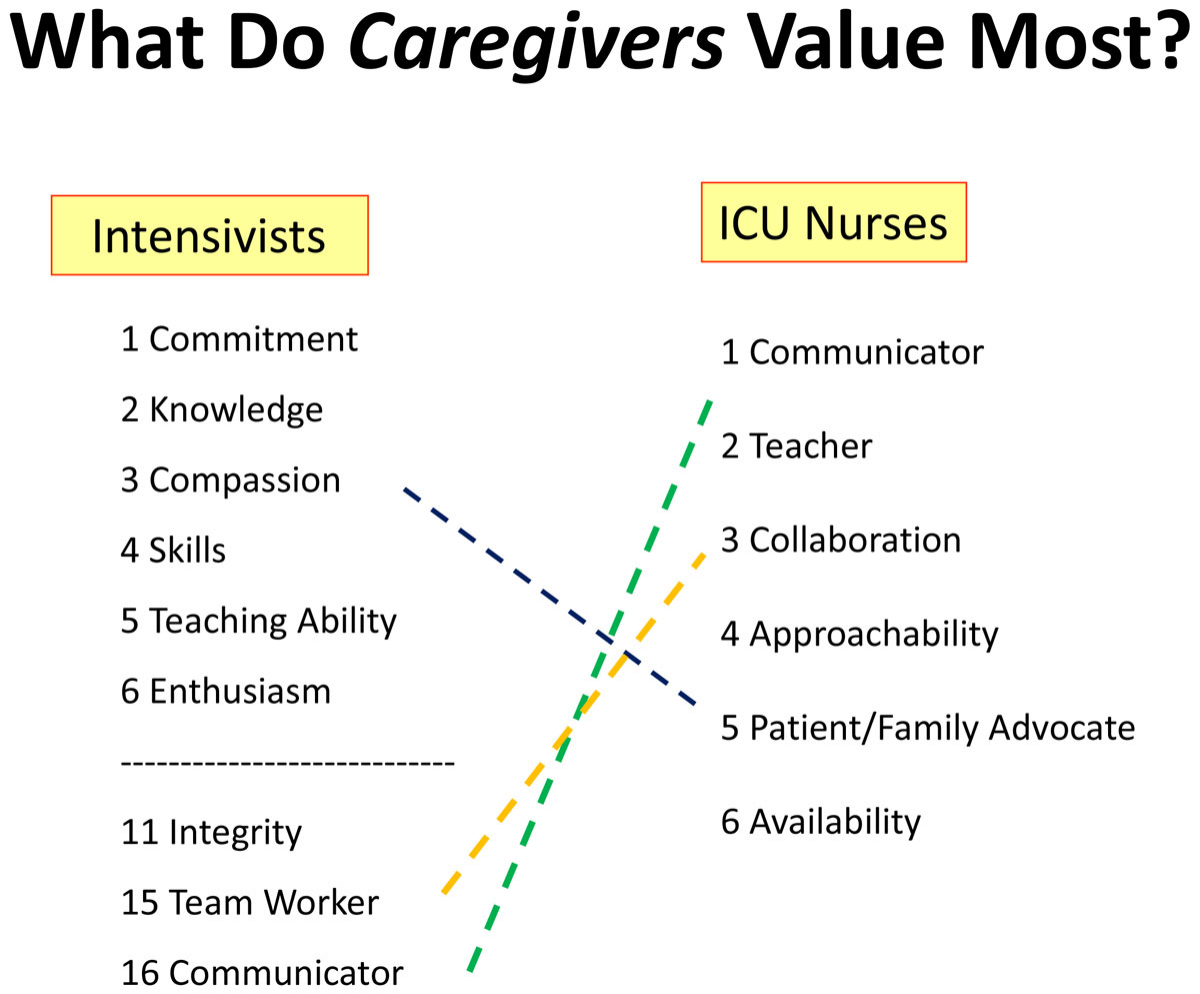
What do *caregivers *value most?

Effort must be exerted at the bedside to work closely with others toward the common goal of patient stabilization and recovery. As the leader of the ICU team, the physician should encourage delegation, communication, and mutual support, showing genuine respect for colleagues, patients, and families alike. A key connection point for all of these stakeholders is the nurse. By continual proximity to the patient, the nurse is well positioned to understand diverse problems, intervention effects, and disease progression and therefore to link all concerned individuals. In a recent survey of elite physicians asked to rank the top attributes of excellence among intensive care physicians, commitment and compassion joined knowledge, teaching ability, and leadership skills as the characteristics most admired in their working colleagues [18]. It is interesting to note that ICU nurses appear to value communication, teaching, collaboration, approachability, patient family advocacy, and availability even more highly than technical knowledge of the discipline (Figure 2). Paradoxically, these highly valued humanistic and professional characteristics have not received sufficient emphasis in modern medical education.

From the patient's perspective, an excellent and trusted physician projects truthfulness, approachability, and a caring attitude. Perhaps most importantly, the outstanding practitioner commits extensive face-to-face connection time. Although not always justified, competence and thoroughness of the physician are usually assumed on the basis of the doctor's presumed knowledge base, experience, and certification. The valued ICU practitioner understands that the patient's life is akin to one long movie, not just the single limited frame of the critical care experience. Yet the ICU environment predisposes to disconnection from the patient on both ends of the stay--sudden and usually unanticipated need for specialized service at entry, limited awareness during treatment, and expedient discharge to the accepting service. To continue the movie metaphor, we try to splice together the film on either side of the ICU experience, ideally snipping it out of the patient's life experience. Understanding and respecting the preadmission life of the patient helps distinguish the truly excellent physician from the one who is merely competent.

Because ICU physicians encounter complex and rapidly changing problems, it is vital to continually reassess and adjust management decisions in a short-loop feedback cycle. When certainty is not assured, an empirical approach with midcourse adjustments and corrections is essential to positive outcomes. The patient's condition is influenced by disease complexity, by stage severity, and by timing of management interventions; therefore, an intervention's value or hazard may be altered by the rapid pace of disease evolution. No matter how confident the physician may be of his or her management, some degree of uncertainty is always present. The skillful physician stays vigilant and a little bit nervous about making decisions. The wise ICU specialist follows trends of his/her patient's response and disease course and not simply the current status, interrupting, modifying, or introducing new therapy as the trend dictates. In this process, the EMR must be used carefully [14,15]. The EMR is extremely helpful for document sharing and retrieval, providing ubiquitous access and the ability to consult medical references at short notice. The EMR, however, can prove detrimental by containing erroneous data, consuming excessive time for data entry, or encouraging detachment from the patient and from the other members of the caregiving team.

In developing relationships with the patient and family it must be remembered that both usually feel vulnerable and diminished by the illness. The natural status gap between doctor and patient--rooted in the physician's authoritative position within the healthcare delivery mechanism, in his/her superior understanding of likely events, and in his/her control of management decisions--needs closure. To bridge the divide, the physician should express a sincere interest in the patient's current or prior occupation, hobbies, and opinions. The physician must remain honest and open, explaining the logic of medical thinking regarding management steps, the level of certainty regarding outcome, and the contingency plan in the event that planned tests or responses do not go as expected. Light and respectful humor helps level the playing field. The caregiving team must recognize and acknowledge our limitations as we make a compassionate attempt to overcome the problem, recover prior health status, and make things right again. Early rehabilitation and mobilization have received appropriate emphasis. Conversely, the recent emergence of palliative care teams that comanage patients who cannot meaningfully recover or will face significant loss of health status is a highly valued contribution to modern practice [19]. Because mentoring and role modeling are essential to the transmission of professional principles and mores, we must invest more time in our next generation than today's practice patterns afford.

Certain principles of intensive care have been modified in recent years. There has been a beneficial movement away from the attempt to re-establish physiologic normality and toward adapting the patient to what usually proves to be a protracted climb back towards functionality. We have learned hard but very important lessons, which include: adopting normal targets at which to direct treatment for patients with limited reserve entails serious and unnecessary risk; because many patients are destined to die despite our best efforts, we should use discretion in what we offer--the best course is often palliation and comfort only; mistakes occur frequently when care is complex, rushed, disorganized, and poorly coordinated; aggressive therapies have the potential for harm, both obvious and covert--despite our best efforts, recovery may be slow and incomplete, often culminating in chronic critical illness; and gradual and less often proves better than aggressive and more, as we have found repeatedly regarding ventilating pressures [20,21], intravenous fluids, vasopressors, antibiotics, blood products [22], electrolyte replacement, sedation, and bed rest [23].

Stages of critical illness proceed from initial rescue to stabilization, strengthening, and recovery. Whereas it is uncontestable that vigorous rescue attempts are needed in the very first phase of acute critical illness and that we are becoming better at achieving cardiopulmonary stabilization, sustaining these interventions may have lingering after-effects that thwart recovery. Cognitive outcomes after ARDS and sepsis, for example, have been slow to recover [5]. High-level functions--such as mental processing speed, memory, executive functioning, attentiveness, and IQ--may be impaired for as long as two years after life-threatening illness [5,23]. We must question whether we are doing too much for too long and could do better in timing our "therapeutic flip" from early rescue to the recovery phase. Early rehabilitation, although difficult in many cases, should be preferred to bed rest once rescue has been completed. While restoring muscular function and promoting earlier mobilization is supported widely [6], some authors believe that once stabilized we should consider strengthening our patients by accepting abnormal but tolerable targets [24,25], and gradually and methodically reloading appropriate patients in other aspects of treatment (e.g., permissive hypoxemia) [26]. Doing so will require us to adopt revised targets for blood gases, blood pressure, and transfusion thresholds. While we know relatively little regarding adaptive response to serious illness, healthy humans deal impressively well with the disordered physiology of high altitude and stresses of extreme exercise [27].

Many questions remain before we can feel confident that we are doing the best for our critically ill patients. Extracorporeal gas exchange holds great promise to avoid the need for invasive mechanical ventilation with its attendant risks for infection, ventilator-induced lung injury (VILI), and oversedation. In our enthusiasm to encourage adaptation and re-strengthening of the patient recovering from catastrophic illness, we must not overload the patient by withdrawing too much of our supportive care too soon. We are on the verge of a new era in molecular medicine in which we will more precisely understand the patient's disease, vulnerabilities, and reserve. For the specific patient under consideration, newer technologies will better monitor organ function, physiological tolerance, and clinical progress [28]. Some believe (with good justification) that these coming developments, coupled by guidance from "big data' analysis and real-time data crunching [29,30], will usher in an era of "personalized" or "precision" medicine [10]. Clearly our ability to rapidly evaluate the genome and its products--transcripts, proteins, and biometabolic mediators--will help [28], as will improving knowledge regarding the disrupted chronobiology of critical illness [31]. More efficient and thoughtful architectures for RCTs (such as adaptive design) will help put our new concepts to the final test [32].

**Figure 3:**
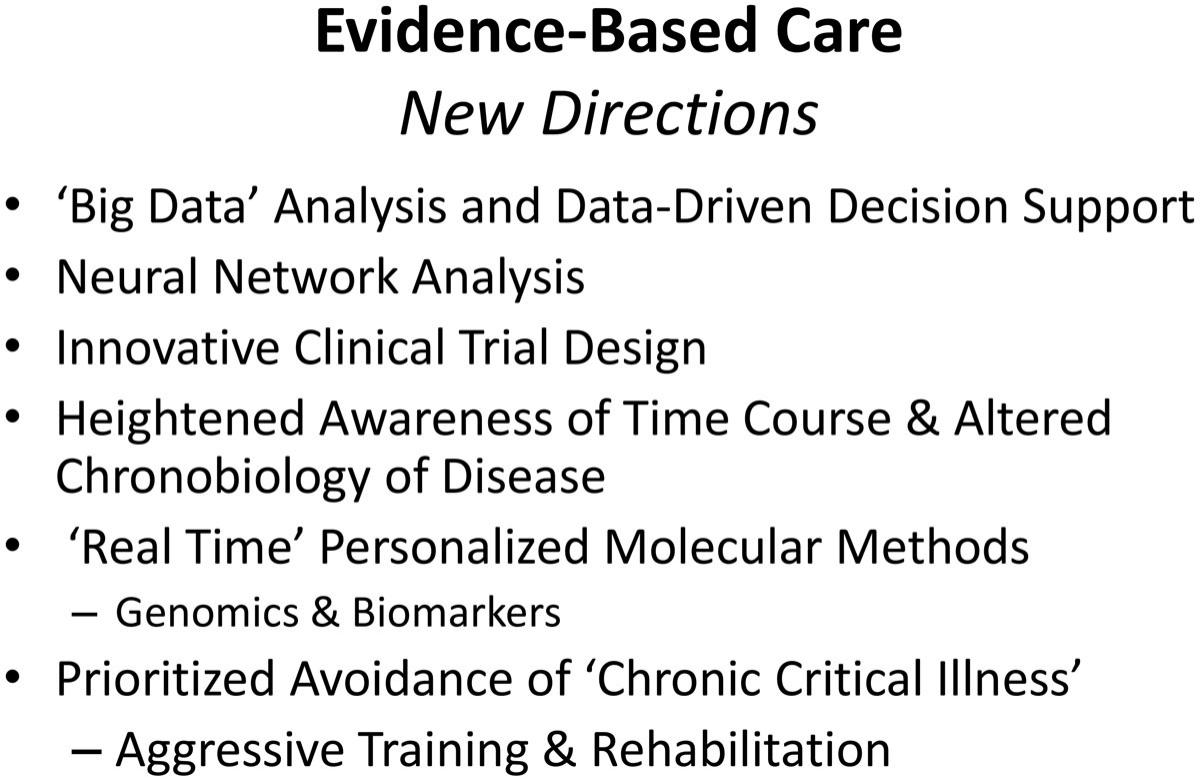
**Evidence-based care: new directions**.

New directions in evidence-based care were suggested recently by experts in intensive care research at an extended roundtable forum conducted in Brussels in 2014 [33] (Figure 3). Among the important ideas shared at the conference were that large data capability for analysis and data-driven decision support hold major promise for improving our bedside care. Neural networks, innovative trial design, and open databases should help to unravel the complexity of the diseases that we encounter. Heightened awareness of the time course and altered chronobiology of disease and 'real-time' personalized molecular methods for detecting biomarkers of genomic expression at the bedside should also help us precisely target our management interventions.

Such sophisticated approaches are not accessible to all hospitals. However, all caregivers can embrace innovations that deliver more "connected" care and that energize and humanize our working conditions. We need to reorganize our care delivery systems to improve efficiency and the environments in which we work. Timely intervention and withdrawal of support is fundamental to intelligent and humane care delivery in the ICU. We must renew and teach the core elements of our profession--physiology, responsibility, and empathy--and thereby restore vital connections to patient and family. Our systems should better adjust delivery capacity to workload so as to develop flexibility, consistency, and reduced stress encountered by the caregiving team.

Training mid-level providers of intensive care, such as critical care trained nurse practitioner and physician assistants, should be a priority of our discipline [34]. Such key personnel link the patient to the physician while freeing up the physician's time. The critical care trained nurse practitioner or physician's assistant can document effectively and connect with family, other physicians, pharmacists, respiratory therapists, medical trainees, and other ancillary personnel. Wise use of effective communications and translational technologies such as voice to text documentation, video links to the bedside, and pooled EMRs will help in the delivery of timely and consistent care. In many hospitals and clinics, medical scribes help spare the physician the onerous duty of documentation, thereby freeing up valued time to enable reconnection. Perhaps with use of such innovations we can return to the nourishing roots of critical care, which are deep physiological understanding, dedication, and empathy. In striving to become a better intensivist, three vital watchwords are connection, communication, and compassion. Together they sum to commitment.

## Summary

Developments in recent years have placed powerful new tools at the disposal of medicine in general, and of critical care in particular. These have great potential to revise and perfect our current approaches, which have succeeded impressively in certain respects and failed impressively in others. The digital revolution has swept into practice and into the research arena, with major potential to improve progress in both arenas. Healing, however, requires not only technical proficiency, but also personal connection and commitment to the venerable traditions of our profession. Re-tooling critical care for the future generations of caregivers requires something old--empathetic connection--as well as the exciting newer technologies of our science and practice.

## Abbreviations

ARDS, Acute respiratory distress syndrome; EBM, Evidence-based medicine; EHR, Electronic health records; EMR, Electronic medical record; RCT, Randomized clinical trial; VILI, Ventilator-induced lung injury.

## Competing interests

The author declares that he has no competing interests.

## Notes

This article has been published as part of *Critical Care *Volume 19 Supplement 3, 2015: Future of Critical Care Medicine (FCCM) 2014. The full contents of the supplement are available online at http://www.ccforum.com/supplements/19/S3. Publication of this supplement was supported by Fresenius Kabi.
